# Convention of Delegates from Western Medical Schools

**Published:** 1848

**Authors:** 


### ARTICLE II.

### CONVENTION OF DELEGATES FROM WESTERN MEDICAL SCHOOLS.

It is suggested in the Ohio Medical and Surgical Journal that a convention of this character be held at Cincinnati on the last Tuesday in April next, for the purpose of securing a uniformity of action amongst the schools.

The points the editor hopes to hear discussed amongst others are, the propriety of extendng the lecture term uniformly to five months; qualifications, both preliminary and professional, for a degree; and the general subjects of lecture fees. The editor adds, "Let the schools in good faith unite upon a platform, and they will all flourish eqnally well, and advance more rapidly the true interests of the profession and the public."

We like the proposition well, and would say on behalf of the faculty of Rush Medical College that if such a convention is agreed upon generally, at that or any other time, or place, they will be represented. E.

